# Retrospective Review of MET Gene Mutations

**DOI:** 10.18632/oncoscience.161

**Published:** 2015-05-14

**Authors:** Maryam Zenali, James deKay, Zesheng Liu, Stanley Hamilton, Zhuang Zuo, Xinyan Lu, Rania Bakkar, Gordon Mills, Russell Broaddus

**Affiliations:** ^1^ Department of Pathology and Laboratory Medicine, University of Texas MD Anderson Cancer Center, Houston, Texas; ^2^ Department of Pathology and Laboratory Medicine, University of Vermont Medical Center, Burlington, Vermont; ^3^ Institute of Personalized Cancer Therapy, University of Texas MD Anderson Cancer Center, Houston, Texas; ^4^ Department of Molecular Pathology, University of Texas MD Anderson Cancer Center, Houston, Texas; ^5^ Department of Hematopathology, University of Texas MD Anderson Cancer Center, Houston, Texas; ^6^ Department of Pathology, University of New Mexico, Albuquerque, New Mexico

**Keywords:** C-MET, co-mutations, FISH, targeted therapy

## Abstract

C-MET proto-oncogene is a tyrosine kinase situated on chromosome 7. C-MET and its ligand hepatocyte growth factor/scatter factor (HGF/SF) play a role in proliferation, differentiation and organ development. C-MET genetic aberrations are found associated with driving tumorigenesis. In this retrospective study, we reviewed molecular analysis data gathered from a cancer institute during a two-year period (2010-2012). Upon detection of tumors harboring c-MET mutations, we determined the status of the other mutations tested and evaluated c-MET expression by fluorescent in-situ hybridization (FISH). Our search resulted in identification of 134 c-MET mutations, 44% of which had mutations of at least one of the other genes tested. No c-MET expression aberrancy was detected in this subset by FISH. Survival amongst the patients with surgically resected metastatic colorectal cancers (CRC) was slightly better in those with only a c-MET mutation compared to those with no mutation detected, although the difference was not statistically significant. When c-MET inhibition becomes an integrated part of chemotherapy practice, our observed frequency of co-mutations will be an argument for utilizing c-MET targeted treatment in combination with other targeted drugs and therapeutic strategies. Larger studies can aid to further parse out c-MET prognostic and therapeutic significance.

## INTRODUCTION

C-MET tyrosine kinase was first discovered in the1980s [1]. C-MET and its ligand HGF/SF are essential cellular components during embryogenesis, morphogenesis, and development of organs such as liver, skeletal muscle, and placenta. C-MET aberrations at the genetic level or post-translational overexpression of the receptor have been studied in many tumors; association of these alterations with oncogenesis and tumor progression has been proposed [1].

C-MET, as detailed in a study by Ma *et. al*, contains 3 main structural domains: an extracellular Sema domain (Exon 2), a juxtamembrane domain (Exons 14/15) and a tyrosine kinase domain where many of the c-MET mutations have been reported [2]. Activation of c-MET is achieved via HGF/SF binding which results in a variety of actions depending on the cellular context, varying from proliferation, motility and differentiation in wild type cells to invasion and metastasis in neoplastic cells. In tyrosine kinase pathways where c-MET is a component, ligand binding precedes receptor dimerization. Mutations in these or associated genes circumvent the appropriate ligand dependent regulation. C-MET activation can occur by amplification/overexpression, activating point mutations, paracrine signaling or over expression of its ligand hepatocyte growth factor/scatter factor [1–4].

C-Met signaling has been implicated in a variety of malignancies, including gastrointestinal, genitourinary, breast, gynecologic, pulmonary, head and neck cancers and melanoma. Its genetic aberrations have been reported in a small percentage of this wide variety of tumors [1,5]. Certain c-MET mutations are found to be germline with unknown transforming activity while others are implicated in driving neoplasia.

Availability HGF/SF-MET inhibitors with a range of potencies and specificities has provided a base for assessing their therapeutic value, and initial clinical trials have shown benefit from these treatments in several tumor types. Since c-MET signaling converges with a multitude of pathways, it can lead to activation of several downstream effectors [5]. This diversity substantiates the efforts of c-MET targeting, but biochemical crosstalk between pathways with potential neoplastic rescue process argues for combination chemotherapy.

In this retrospective study we reviewed molecular diagnostic data resulting from a series of mutational analysis during the 2010-2012 period in a cancer center institution. The primary objective was to gain insight into patterns of c-MET mutations and patterns and frequency of co- mutations. We also compared survival outcome in metastatic colorectal adenocarcinoma between those harboring only c-MET mutations as opposed to those with no mutations detected on a 14-gene test.

## MATERIALS AND METHODS

We reviewed the molecular analysis report from over 2000 solid tumors undergone mutation testing at MD Anderson during 2010-2012. Cases (except a small subset of primary brain tumors) were predominantly high stage solid tumors where the mutational analysis was performed to assess for considerations of targeted additional therapy. These tests included a custom designed mutation analysis panel as described previously (Leuk Lymphoma 54(1):138-44; 2013). This quantitative primer-extension MALDI-TOF assay (PE-MALDI) interrogated a total of 88 hotspot mutations in 11-genes (*AKT-1, BRAF, GNAQ, GNAS, IDH1, IDH2, KRAS, MET, NRAS, PIK3CA, RET*). Mutation detection was carried out utilizing a 9-well based 11-gene mutation hotspot screen developed by our laboratory using Massarray® platform (Sequenom, San Diego, CA). Briefly, DNA was PCR amplified and subjected to single base-primer extension using iPLEX Gold kit and analyzed on MALDI-TOF mass spectrometry (Sequenom, San Diego, CA). The regions of interest were PCR amplified using 10ng of DNA per well. Subsequently PCR products were treated with shrimp alkaline phosphatase to dephosphorylate unincorporated nucleotides followed by locus specific single base extension with mass modified di-deoxy nucleotides (ddNTP).

Primers for both PCR amplification and single-base extension were designed by Sequenom's MassARRAY Designer software and obtained from Integrated DNA Technologies (IDT, Coralville, IA). The mass of the products of single-base extension were analyzed by MALDI-TOF for SNP detection. In addition, the exons 18 to 21 of the *EGFR* gene, exons 11, 13, and 17 of the *KIT* gene and in a subset of cases, the exons 4 to 9 of the *TP53* gene were also analyzed in each sample by Sanger sequencing. In all cases, DNA was obtained from unstained formalin fixed paraffin embedded (FFPE) tissue sections using the PicoPure DNA Extraction Kit (Life Technologies, Grand Island, NY) following manufactures instructions. In the c-MET mutated subset, patient's demographics, tumor types and stage, SNP patterns, their distribution amongst tumor types, secondary tumors and incidences of co-mutations were recorded (Tables 1-3). Metastatic CRC was the largest tumor category harboring c-MET mutation, in which we compared survival outcome of patients with only c-MET mutation to those with no detectable mutation using Kaplan Meier method and difference compared by log-rank. In the same subset of patients we evaluated whether c-MET mutational status showed associations with age and/or gender by logistic regression analysis.

**Table 1:** Tumor types, patient demographics and c-MET mutation patterns

Tumor Type(with c-MET mutation)	Other Tumors in These Patients	Patients' Age/Sex	Tumor Stage	MutationExon(s)	Mutation Codon(s)/aa Change(s)
Colorectal Adenoca. n= 43 % mutated: 9.5%	Melanoma × 1 Prostatic Ca × 1	Median age: 59 Avg age: 56.4 M/F= 28/15	IV: 28 III: 11 II: 4	2 14 14 19 19	N375S × 19/Asn to Ser T1010I × 18/Thr to Ile R988C × 4/Arg to Cys Y1253D × 1/Tyr to Asp Y1248H × 1/Tyr to His
Melanoma n=14 % mutated: 5.5%	BCC &SCC × 4 Dysplastic nevus × 2 PTC × 1 Prostatic Ca × 1	Median age: 62 Avg age: 59 M/F= 10/4	IV: 13 III: 1	2 14 14	N375S × 10/Asn to Ser R988C × 2/Arg to Cys T1010I × 3/Thr to Ile * one tumor had 2 cMET mutations
Gastric Adenoca. n=2 % mutated: 25%		51 y/o F & 69 y/o F	III: 1 II: 1	2	N375S × 2/Asn to Ser
Appendiceal Adenoca. n=2 % mutated: 13%		55 y/o F & 33 y/o F	IV: 2	2 14	N375S × 1/Asn to Ser R988C × 1/Arg to Cys
Hepatocellular Ca n=2 % mutated: 17%		62 y/o M & 72 y/o M	N/A	2 14	N375S × 1/Asn to Ser R988C × 1/Arg to Cys
Duodenal Adenoca. n=1 % mutated: 7%		44 y/o F	IV: 1	2	N375S × 1/Asn to Ser
Pancreatic Adenoca. n=1 % mutated: 8.3%		64 y/o M	IV: 1	2	N375S × 1/Asn to Ser
Lung Adenoca. n=8 % mutated: 4.8%	SCC × 1 Prostatic Ca × 1	Avg age: 64 Median age: 65 M/F: 4/4	IV: 7 N/A: 1	2 14 14	N375S × 6/Asn to Ser R988C × 1/Arg to Cys T1010I × 1/Thr to Ile
Thyroid Papillary Ca n=3 % mutated: 12%	Lung Adenoca. × 1	Avg age: 68 Median age: 65 M/F: N/A	IV: 3	2	N375S × 3/Asn to Ser
Thyroid Medullary Ca n=1 % mutated: 50%		60 y/o M	N/A	14	T1010I × 1/Thr to Ile
Ewing Sarcoma n=1 % mutated: 33%		25 y/o F	N/A	14	T1010I × 1/Thr to Ile
Prostatic Adenoca. n=1 % mutated: 10%		67 y/o M	IV	2	N375S × 1/Asn to Ser
Squamous Cell Ca of H&N and Cervix n=6 % mutated: 6.3%		Median age: 57 Avg age: 58 M/F: 5/1	IV: 4 N/A: 2	2 14 14	N375S × 2/Asn to Ser R988C × 2/Arg to Cys T1010I × 2/Thr to Ile
Renal Cell Ca n=1 % mutated: 25%		65 y/o M	IV	2	N375S × 1/Asn to Ser
Pheochromocytoma &Composite Pheochromocytoma n=2 % mutated: 100%		71 F & 48 y/o F	IV	2 14	N375S × 1/Asn to Ser R988C × 1/Arg to Cys
Ovarian Serous Ca n=4 % mutated: 8.3%	Breast Ca	Median age: 56 Avg age: 56.2 4F	III: 3 IV: 1	2 14	N375S × 2/Asn to Ser T1010I × 2/Thr to Ile
Ovarian Clear Cell Ca n=1 % mutated: 11%	BCC	53 y/o F	IV	2	N375S × 1/Asn to Ser
Ovarian Mixed Ca n=1 % mutated: 50%		61 y/o F	N/A	14	T1010I × 1/Thr to Ile
Peritoneal Serous Ca n=1 % mutated: 50%		56 y/o F	III	14	T1010I × 1/Thr to Ile
Breast Ductal Adenoca. n=12 % mutated: 20%		Median age: 55 Avg age: 55.31 2F	IV: 7 III: 1 II: 1 N/A: 3	2	N375S × 9/Asn to Ser T1010I × 3/Thr to Ile
Uterine Leiomyosarcoma n=2 % mutated: 22%		54 y/o F & 56 y/o F	IV: 1 I: 1	2	N375S × 1/Asn to Ser T1010I × 1/Thr to Ile
Uterine Endometrioid Adenoca. n=3 % mutated: 13%	Breast Ca. × 1 Colonic Adenoca. × 1	Median age: 63 Avg age: 66.6 3 F	IV: 2 II: 1	2	N375S × 2/Asn to Ser T1010I × 1/Thr to Ile
Uterine MMMT n=1 % mutated: 25%		59 y/o F	N/A	2	N375S × 1/Asn to Ser
Glioblastoma n=5 % mutated: 16%	AML × 1 BCC × 1	Median: 59 y/o Avg age: 56.6 M/F: 3/2		2	N375S × 5/Asn to Ser
Anaplastic Glioma n=2 % mutated: 66%		42 y/o M & 45 y/o M		2	N375S × 2/Asn to Ser
Oligodendroglioma n=1 % mutated: 50%		33 y/o F		14	T1010I × 1/Thr to Ile
Desmoplastic Small Round Cell Tumor n=1 % mutated: 14%		17 y/o M	N/A	2	N375S × 1/Asn to Ser
Squamous Cell Ca of Rectum n=1 % mutated: 25%		N/A	N/A	2	N375S × 1/Asn to Ser
Salivary Gland Can=2% mutated: 10%	PTC × 1	48 y/o F & 73 y/o M	IV: 1 N/A: 1	2	N375S × 2/Asn to Ser
Heart Angiosarcoma n=2 % mutated: 50%		59 y/o M & 31 y/o F	N/A	2 14	N375S × 1/Asn to Ser T1010I × 1/Thr to Ile
GIST n=2 % mutated: N/A		57 y/o M & 67 y/o M	IV: 1 N/A: 1	14 14	T1010I × 1/Thr to Ile R988C × 1/Arg to Cys
Invasive Thymoma n=1 % mutated: 50%		N/A	IV	2	N375S × 1/Asn to Ser
Spindle Sarcoma NOS n=2 % mutated: 16%		61 y/o M & 69 y/o M	N/A	2	N375S × 2/Asn to Ser
Malignant Mesothelioma n=1 % mutated: 33%		59 y/o M	N/A	2	N375S × 1/Asn to Ser

Abbreviations: y/o - year old, avg- average, M- male, F- female, Adenoca.- Adenocarcinoma, Ca- Carcinoma, SCC- Squamous cell carcinoma, BCC- Basal cell carcinoma, PTC- Papillary thyroid cancer, AML- Acute myeloid leukemia, MMMT - Malignant mixed mullerian tumor, GIST - Gastrointestinal stromal tumor, and N/A – Not available

**Table 2:** Summary of incidence of c-MET point mutations with corresponding co-mutations in the studied tumors

Co-Mutations	N375S	R988C	T1010I	Y1248H	Y1253D
P53	4	0	4	0	1
KRAS	13	5	9	0	1
NRAS	3	2	0	0	0
BRAF	8	2	3	0	0
PIK3CA	5	3	1	0	0
IDH1	1	0	1	0	0
EGFR	1	0	0	0	0
AKT	0	0	2	0	0
KIT	0	1	1	0	0

**Table 3:** Summary of tumor types and their corresponding c-MET point mutations and co-mutations

Tumor Type(s)	c-MET Mutation(s)	Co-mutation(s)
Colorectal Adenocarcinoma	N375S, R988C, T1010I, Y1248H, Y1253D	P53, KRAS, PI3K, BRAF, IDH1, AKT
Melanoma	N375S, T1010I, R988C	NRAS, BRAF
Lung Adenocarcinoma	N375S, R988C, T1010I	EGFR, KRAS, BRAF, P53, NRAS
Thyroid Carcinoma	N375S (PTC), T1010I (Medullary carcinoma)*	BRAF, PI3K, P53*
Ovarian Carcinoma	N375S, T1010I	P53, KRAS
Uterine Endometriod Carcinoma	T1010I, N375S	AKT, KRAS
Breast Adenocarcinoma	N375S	PI3K
Squamous Cell Carcinoma	N375S, R988C, T1010I	PI3K
Prostatic Adenocarcinoma	N375S	P53
Appendiceal Adenocarcinoma	N375S, R988C	KRAS
Duodenal & Pancreatic Adenocarcinoma	N375S	KRAS
Heart Angiosarcoma	T1010I, N375S	KRAS
Gastrointestinal Stromal Tumor	T1010I, R988C	KIT
Oligodendroglioma	T1010I	IDH1

Additionally, we reviewed the c-MET FISH results when available, to examine whether there is an association of MET amplification with presence of genetic aberration. MET FISH was performed in the clinical cytogenetics diagnostic lab at MD Anderson Cancer center, following the standard clinical procedure established in the laboratory. In brief, glass slides from formalin-fixed paraffin embedded tissue (FFPE) are pre-treated and hybridized with the MET and chromosome 7-centromere FISH probe mixture (Poseidon/Empire Genomics, Buffalo, NY). FISH results were scored and reviewed on a fluorescence microscope. A total of 60 cells were scored. MET amplification was considered positive if the ratio of average MET to average of CEN 7 was equal or greater than 2.0 or more than 20 copies of MET signals with clusters noted in >10% of the cells.

## RESULTS

Upon review of the molecular data, we found 134 c-MET mutations, involving 133 tumors (2010-2012), which correspond to 6-7% of the total number of tumors. 5 different c-MET point mutations were detected by DNA sequencing (Table 1). The majority of mutations were observed in codon 375. 81 cases had Asn to Ser mutation at codon 375 (N375S), corresponding to 60%. 38 cases had a Thr to Ile mutation at codon 1010 (T1010I), corresponding to 28%. 13 cases had an Arg to Cys at codon 988 (R988C), corresponding to 10%. One case had a Tyr to His mutation at codon 1248 (Y1248H), corresponding to 0.7%, and one had a Tyr to Asp at codon 1253 (Y1253D), corresponding to 0.7%. One case of melanoma (Lentigo Maligna type/stage IV disease) had 2 simultaneous c-MET mutations (N375S and T1010I). FISH assay for c-MET gene copy number amplification was carried in 46% of the c-MET mutated (62/134), all of which were negative.

44% (59 of 134) cases with c-MET mutation had mutations of at least one of the other 13 genes in the tested (10 additional genes by Sequenom and 3 by Sangers) with 9 % (12 of 134) having 2 or more co-mutations. These comutated genes included p53, KRAS, NRAS, BRAF, PIK3CA, IDH1 KIT, AKT and EGFR, the distribution of which corresponded to trends generally associated with each tumor type. Save for the one case with Y1253D aberration and co-mutations (1/1=100%), the highest rate of co-mutations was observed with R988C aberration, corresponding to 76% (10/13). The next highest co-mutation rate was with T1010I, corresponding to 37% (14/38) closely followed by N375S at 35% (29/81). No co-mutation was clustered with codon Y1248H. This patient also had history of squamous cell carcinoma and prostatic adenocarcinoma.

**Figure 1:**
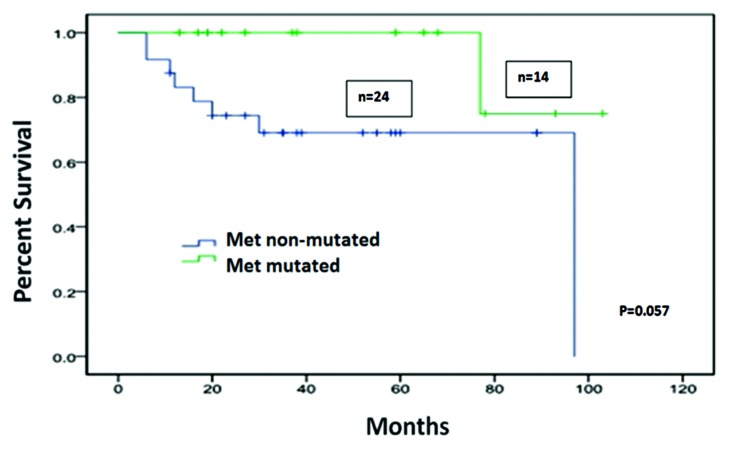
Kaplan Meier Graph of survival comparision between patients with metastatic CRC and only c-MET mutation vs. those who no mutation detected on the molecular test panel

In cases with metastatic, surgically resected colorectal percentages were detected: c-MET 9.5%, KRAS 43%, PIK3CA 18%, NRAS 4%, and BRAF 8%. The remaining mutations included P53, EGFR, KIT, and IDH mutations.3% of tumors had no mutation detected on analysis. Neither the c-MET mutated nor the subset of cases with no mutation detected showed overexpression of c-MET by FISH, and targeted c-MET treatment was not utilized in their treatment. Those whose sole mutation detected on the panel was a c-MET mutation (n=14) had a better survival compared to subjects with no mutations detected in the analysis (n=24). However, this difference was only marginally significant (p=0.057), which may be due to limited sample size. Results are summarized in tables 1-3 and Figure 1 (Kaplan Meier curve). In cases with metastatic CRC, logistic regression analysis of age and gender against c-MET mutational status was negative for any significant association (p: 0.88 and p: 0.56 respectively).

## DISCUSSION

C-MET receptor tyrosine kinase and its physiological ligands HGF/SF are associated with diverse cellular response upon activation. Ligand mediated dimerization triggers phosphorylation of c-MET and response of cytoplasmic effector proteins including GAB1, GRB2, phospholipase C and SRC. C-MET is intimately associated with pathways including RAS-MAPK, PI3K-AKT and Wnt [5].

The role of c-MET in tumorigenesis through genetic aberration, up regulation of cellular cross talk, and roles in metastasis and activation of cancer stem cells is an area of ongoing research. The evidence for it playing a role in oncogenesis is mounting as not only its somatic mutations have been found in many cancers, germ line c-MET point mutations are present in subset of tumors such as papillary renal cell carcinoma. In parallel a subset of genetic mutations are found as polymorphic variants. Although in our study we did not find upregulation in MET gene expression by FISH analysis, we did detect missense point mutations that through their altered gene product can change the functionality of the c-MET receptor.

The most commonly identified mutation in our series was the N375S of the extracellular SEMA domain. This aberration results in the medium sized polar amino acid asparagine being replaced by the smaller polar amino acid serine and has been found as a germ line mutation in a cohort of patients with non-small cell lung cancer (NSCLC) [6]. A later study found this mutation at a higher rate in the normal healthy controls than in the tumors indicating is a germline polymorphism rather than a cancer driving aberration [7]. Similar findings are indicated by Greenman *et. al.* to find N375S as a passenger and not a driver mutation [8]. These studies clearly cast doubt as to its role in tumorogenesis. The N375S mutation has been found to result in a c-MET receptor which is resistant to inhibition [6]. In comparison, the T1010I mutation is found a more robust target for chemotherapy [9,10]. It is plausible that in a tumor harboring concurrent c-MET aberrations, such as the one melanoma case in our review, the N375S mutation may hinder the efficacy of the c-MET directed treatment. As chemotherapy becomes more sophisticated and personalized medicine gains reality, the implications of mutations such as the N375S mutation can become more relevant in establishing targeted treatments.

Despite evidence supporting the T1010I mutation's importance in oncogenesis, not every study confirms this role. This mutation, in the cytoplasmic juxtamembrane domain, results in the substitution of the medium sized hydrophobic amino acid threonine with the medium sized polar amino acid isoleucine. It has been shown to increase growth factor independent proliferation and motility in vitro in tumor cell lines [9]. Wasenius et. al. found this mutation more frequently in thyroid carcinomas than in the goiter controls [10]. Interestingly, 4 of the 6 mutations were also found to be germline. Yet its significance has been challenged by other studies which report a low incidence of T1010I mutation in both tumors and controls and not resulting in an enhanced c-MET phosphorylation [9,11].

The other c-MET mutation R988C, affecting the cytoplasmic juxtamembrane domain, results in the substitution of the large polar amino acid arginine with the small polar amino acid cysteine. Studies on this mutation are also conflicting as some demonstrate it resulting in gain a function in SCLC cell lines [9,12], while others report its lack of transforming oncogenic capacity and therefore of little significance [11,13].

The two remaining mutations detected are both affecting the kinase domain. The Y1248H mutation results in the substitution of the large non-polar aromatic amino acid tyrosine with the medium sized polar amino acid histidine. It is a known mutation found in papillary renal cell carcinoma and reported to have a transforming ability [14,15]. This mutation is relevant in chemotherapeutic targeting and has been found resistant to the c-MET inhibitor SU11274 [16]. The Y1253D mutation results in the substitution of the large non-polar aromatic amino acid tyrosine for the medium sized polar amino acid aspartic acid. This mutation has been found in nodal metastases of head and neck carcinoma and when introduced into a cell line resulted in a more motile and invasive cell phenotype [17].

The discrepancies between studies on whether a particular mutation is transformative may be explained if the same aberration results in different cell phenotypes when affecting different cell lineages. To support this notion, a recent study indicates that overexpression of c-MET associated with a worse prognosis in esophageal adenocarcinoma and not in head and neck cancers [18]. Despite the controversies regarding functionality of c-MET mutations, more recent and uniformly reported observations are that c-MET mutation in conjunction with other mutations likely confers a more aggressive tumor profile and that c-MET aberration is more prevalent in metastatic/invasive foci compared to the primary tumor [17,18].

Polymorphisms of c-MET which on their own may lack transformative ability may still play a role in promotion of tumor development and metastasis in combination with other mutations. Its relevance may be closely tied to co- mutation rather than the sole effect of this mutation alone. According to Lee et al., activated c-MET has been shown to be a predictor of poor survival when in combination with other mutations in CRC. The authors identified that clustering of activated c-MET in conjunction with PIK3CA mutation predicts a worse survival outcome [19]. The effect of these mutations may depend on whether evolved in germ line or somatic cells, the paracrine milieu in addition to the presence of secondary co-mutations.

As stated previously, a subset of c-MET aberrations can play a role in response to chemotherapy by rendering the receptors resistant to drug therapies targeting c-MET. This would clearly effect the treatment response by decreasing the response to the drug. In EGFR mutated NSCLC, MET amplification has been demonstrated as an escape pathway and a mechanism of EGFR TKI resistance [20]. Also, in patients with c-MET amplified NSCLC, addition of a c-MET inhibitor has been shown to lead to improvement of survival [21]. These findings make it likely that MET inhibitors will be an important addition to combination chemotherapy and also more robust tumor profiling, an important part of patient care.

Current literature empirically suggests that c-MET aberrations (mutations/protein over expression) are more commonly noted in tumor clones with potential to spread. Activating c-MET aberrations have been found selected in association with nodal metastasis or invasive fronds of the primary tumors. This increased prevalence has been theorized to involve c-MET's signaling function in angiogenesis and cell movement [6,20,21]. A higher than expected range of c-MET mutations noted in our review is perhaps in parallel with the notion of clustering of c-MET with metastatic fronds, as greater than 95% of cases in our study were of metastatic tumors.

Diversity of proposed neoplastic mechanisms has led to producing a wealth of inhibitors targeting various aspects of the c-MET pathway. Currently clinical trials are underway involving c-MET and cancers including NSCLC, hepatocellular carcinoma, renal cell carcinoma, cholangiocarcinoma, triple negative breast adenocarcinoma, gastric or esophageal adenocarcinoma, and anaplastic large cell lymphoma, to name a few (http://clinicaltrials.gov/ct2/results?term=met). Most of these trials investigate the effects of c-MET inhibitors, usually in combination with other tyrosine kinase inhibitors or other chemotherapy drugs.

Our study did not find a survival disadvantage for patients with resected metastatic CRC harboring only c-MET mutation compared to those with none, but rather a marginally significant survival improvement. Although the small study population may limit analysis, we did not find a clear-cut adverse prognostic relevance attributable to c-MET mutation alone, when unaccompanied by a known oncogenic mutation. Further studies may aid in establishing this concept.
